# Supplementary material for: Electrospun Poly-L-Lactic Acid Scaffolds Surface-Modified via Reactive Magnetron Sputtering Using Different Mixing Ratios of Nitrogen and Xenon
Source: Polymers (Basel). 2023 Jul 6;15(13):2969. doi: 10.3390/polym15132969 (PMC10347186; doi:10.3390/polym15132969)
Supplement: Supplementary file 1 [file polymers-15-02969-s001.zip › polymers-2454672-supplementary.pdf]

Support Information of: Electrospun Poly-L-Lactic Acid Scaffolds Surface-Modified via Reactive Magnetron Sputtering Using Different Mixing Ratios of Nitrogen and Xenon

Pavel V. Maryin [1], Tuan-Hoang Tran [1], Anastasia A. Evtina [2], Mikhail A. Buldakov [2], Evgeny L. Choinzonov [2], Anna I. Kozelskaya [1], Sven Rutkowski [1*] and Sergei I. Tverdokhlebov [1*]

[1] Weinberg Research Center, School of Nuclear Science & Engineering, National Research Tomsk Polytechnic University, 30, Lenin Avenue, 634050 Tomsk, Russia; pvm5@tpu.ru (P.V.M.); cungbinh9327@gmail.com (T.-H.T.); kozelskayaai@tpu.ru (A.I.K.)

[2] Cancer Research Institute of Tomsk National Research Medical Center of Russian Academy of Sciences, Kooperativni Str. 5, 634050 Tomsk, Russia; frolova_aa@onco.tnimc.ru (A.A.E.); buldakov@oncology.tomsk.ru (M.A.B.); choynzonov@tnimc.ru (E.L.C.)

* Correspondence: rutkowski_s@tpu.ru (S.R.); tverd@tpu.ru (S.I.T.)

1. Supplementary Figures

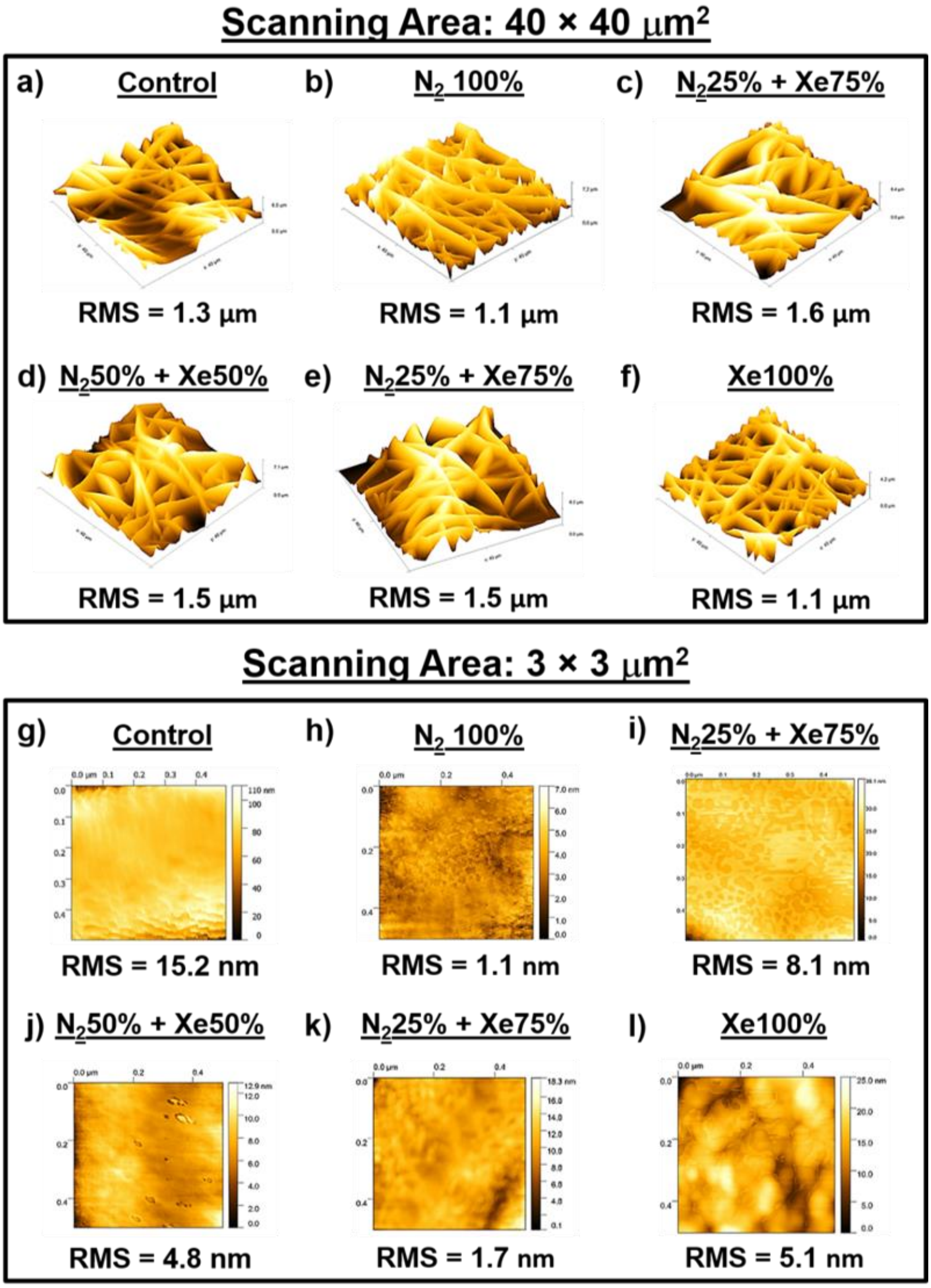

Figure S1. a) – f) AFM micrographs obtained at a scanning area of 40 × 40 μm², and g) – l) AFM micrographs obtained at a scanning area of 3 × 3 μm². Unmodified PLLA scaffold: a), g) control samples; PLLA scaffolds surface-modified in nitrogen and xenon at different mixing ratios: b) and h) N_2 100%, c) and i) Xe75% + $N_2$25%, d) and j) Xe50% + $N_2$50%, e) and k) Xe25% + $N_2$75%, f) and l) Xe 100%.

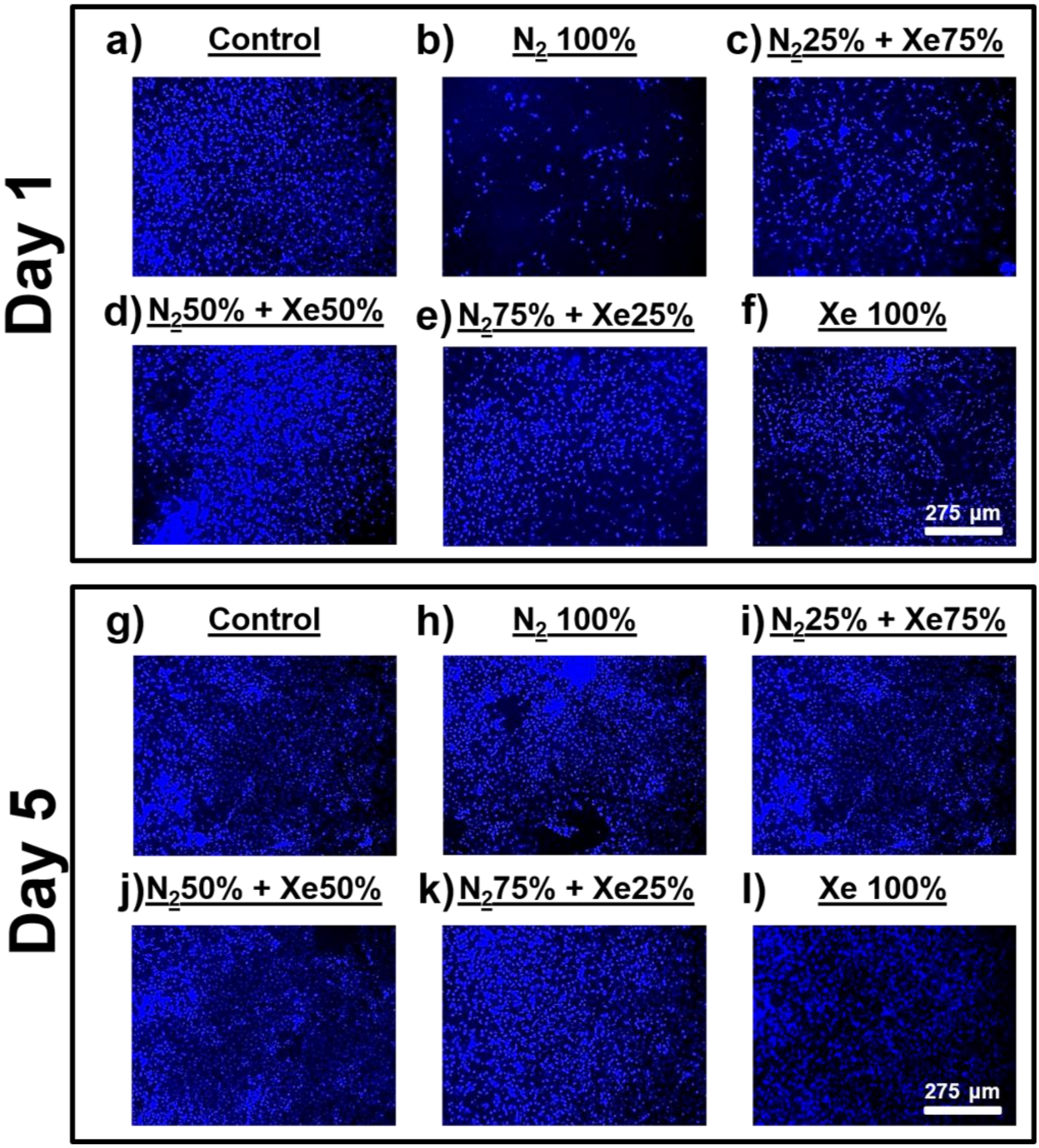

Figure S2. Fluorescence micrographs of HOS cell nuclei on the PLLA scaffold sample surfaces under investigation and surface-modified in a mixture of nitrogen and xenon at different mixing ratios, obtained after 1 day a) – f) and 5 days g) – l) of cell cultivation. Unmodified PLLA scaffold: a), g) – control samples; PLLA scaffolds surface-modified: b) and h) N_2 100%, c) and i) $N_2$25% + Xe75%, d) and j) $N_2$50% + Xe50%, e) and k) $N_2$75% + Xe25%, f) and l) Xe 100%. The scale bars shown are the same for all micrographs (275 μm).

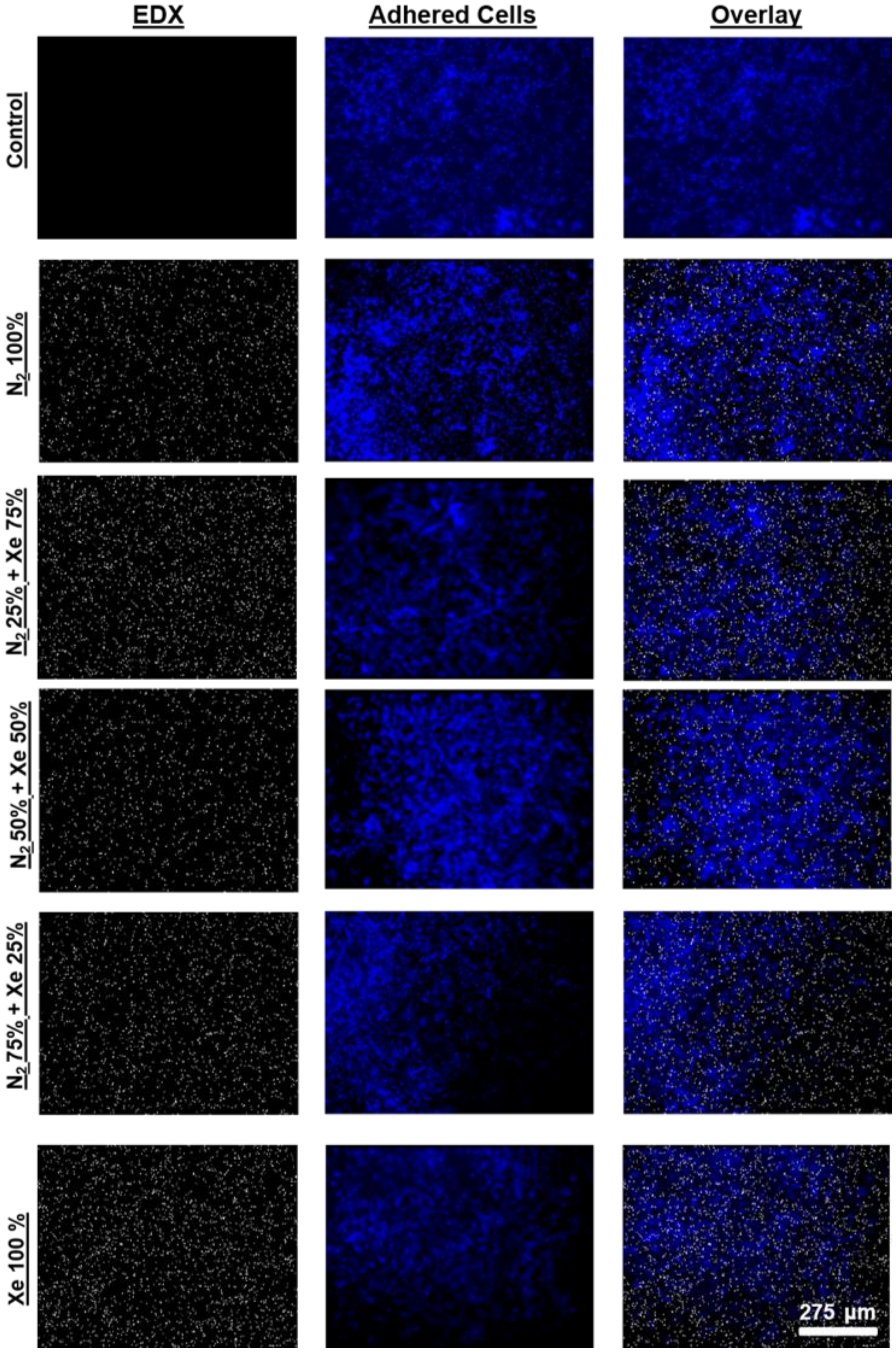

Figure S3. EDX mapping of Ti on surface-modified PLLA scaffolds: left column displays the EDX mapping micrographs of Ti on the scaffold fibers, the middle column shows micrographs cells adhered on the scaffold samples, and the right column demonstrated the overlay of both types of micrographs. The scale bar for each micrograph is 275 μm.

2. Supplementary Table

Table S1. Elemental composition of all PLLA scaffolds under investigation depending on the mixture of working and noble gases obtained by XPS analysis.

Gas Mixture	Element concentration, at%				Ti/N	N/O
	C	O	Ti	N		
Control	73.66 ± 1.47	26.34 ± 0.53	–	–	-	-
N_2 100%	56.26 ± 1.75	29.47 ± 0.62	9.27 ± 0.12	4.14 ± 0.09	1.54	0.17
$N_2$25% + Xe75%	60.65 ± 1.77	27.87 ± 0.64	7.34 ± 0.13	5.00 ± 0.04	1.77	0.15
$N_2$50% + Xe50%	63.30 ± 1.56	26.24 ± 0.57	4.92 ± 0.06	5.54 ± 0.1	0.89	0.21
$N_2$75% + Xe25%	57.53 ± 1.67	28.54 ± 0.58	8.43 ± 0.09	5.49 ± 0.14	1.85	0.19
Xe 100%	57.88 ± 1.68	29.99 ± 0.47	9.93 ± 0.1	2.20 ± 0.06	4.51	0.07
